# Special Issue “Regulatory Mechanism and Network of Abiotic Stress Response in Plants 2.0”

**DOI:** 10.3390/ijms27073215

**Published:** 2026-04-01

**Authors:** Hye-Yeon Seok, Yong-Hwan Moon

**Affiliations:** 1 Institute of Systems Biology, Pusan National University, Busan 46241, Republic of Korea; seokhyeon@pusan.ac.kr; 2 Department of Integrated Biological Science, Pusan National University, Busan 46241, Republic of Korea; 3 Department of Molecular Biology, Pusan National University, Busan 46241, Republic of Korea

## 1. Introduction

During their growth and development, plants are constantly exposed to a wide variety of environmental stresses, which can generally be divided into biotic (e.g., pathogen infection and herbivory) and abiotic stresses (e.g., drought, salinity, extreme temperatures, and nutrient deficiency). Abiotic stresses are key environmental factors that limit plant productivity, influence species distribution, and significantly reduce crop yields and quality. These negative effects are further worsened by global climate change, which is expected to increase the frequency and severity of extreme environmental conditions and, in turn, pose a greater threat to agricultural sustainability and food security [1,2,3,4].

Abiotic stresses affect plant performance at multiple biological levels. At the cellular level, drought and salinity cause osmotic and ionic stresses, often accompanied by oxidative damage and metabolic disruption, while at the whole-plant level, these induced stresses impair growth, development, and reproductive success, ultimately reducing biomass and yield [3,4,5]. As sessile organisms, plants survive under fluctuating conditions by relying on their abilities to perceive environmental signals and activate adaptive responses. At the molecular level, abiotic stress responses are governed by complex regulatory networks that control the expression of stress-inducible genes. Transcriptomic studies have revealed that large sets of genes are activated under stress, yielding proteins that function in both tolerance and signal transduction mechanisms [5,6,7,8,9]. Advances in genomic and post-genomic approaches have further expanded this viewpoint from focusing on individual genes to regarding them as highly interconnected regulatory systems [1,10,11].

A key feature of these stress responses is the integration of abscisic acid (ABA)-dependent and -independent pathways. ABA accumulation under stress induces many responsive genes, while parallel pathways regulate additional gene sets through distinct mechanisms [3,12,13]. Abiotic stress signaling is further mediated by kinase cascades, including sucrose nonfermenting 1-related kinases, calcineurin B-like (CBL)–CBL-interacting protein kinase modules, and mitogen-activated protein kinase, which link stress perception to downstream responses [3,14,15,16,17,18,19]. Additionally, stress sensing involves multiple cellular compartments, in which organelle-derived signals and secondary messengers (e.g., Ca^2+^, reactive oxygen species (ROS), and nitric oxide) coordinate gene expression and adaptive responses [20,21,22,23,24]. Despite these advances, important gaps remain in our understanding of how these regulatory pathways are coordinated across biological scales and how plants integrate multiple simultaneous stresses under natural conditions.

This Special Issue, titled “Regulatory Mechanism and Network of Abiotic Stress-Response in Plants 2.0,” comprises sixteen original research articles and two review articles in which the regulatory mechanisms of plant responses to abiotic stresses such as salinity, drought, anaerobic conditions, cold, heat, and intense light were investigated. The contributions to this Special Issue cover model species such as *Arabidopsis thaliana* (*Arabidopsis*) and *Oryza sativa* (rice) as well as economically important crops such as *Brassica rapa* (filed mustard), *Leymus chinensis* (Chinese rye grass), *Pogostemon cablin* (patchouli), *Chenopodium quinoa* (quinoa), *Nicotiana benthamiana* (benthi), *Solanum lycopersicum* L. (tomato), *Abelmoschus esculentus* (okra), and *Persea americana* Mill. (avocado). These studies used various approaches, ranging from the genome-wide identification of regulatory gene families (including WRKY, *GLABROUS1* enhancer binding protein (GeBP), basic leucine-zipper (bZIP), guanine nucleotide-exchange factor (GEF), Casparian strip integrity factor (CIF), and zinc-finger families) to the functional validation and multi-omics-based integration of transcriptomics and metabolomics. Collectively, these articles offer a comprehensive overview of the influences of regulatory mechanisms and networks on stress adaptation in plants.

## 2. Transcriptional Foundation of Stress-Response Networks

The precise regulation of gene expression is central to the adaptation of plants to abiotic stresses [25,26]. Several contributions to this Special Issue focus on transcription factor families that serve as key regulatory nodes within stress-responsive networks. Genome-wide analyses of the bZIP family in *Arabidopsis*, the RopGEF family in *Brassica rapa*, the CIF family in *Pogostemon cablin*, and the *ZAT* genes of the C2H2 subfamily in quinoa are presented, demonstrating the involvement of these transcription factors within feedback and feedforward regulatory loops in plant responses to drought, osmotic, temperature, and anaerobic stresses (contribution 1–4). Functional studies of WRKY transcription factors, such as LcWRKY40 in *Leymus chinensis* and AeWRKY32 and AeWRKY70 in okra, reveal their roles in mediating responses to drought and salinity (contribution 5–6). These studies demonstrate that rather than acting in isolation, transcription factors coordinate in modules to fine-tune gene expression in response to environmental changes.

In addition to transcription factor activity, post-transcriptional and chromatin-related mechanisms provide additional regulatory layers [27,28]. One study in this Special Issue, looking at miRNA-mediated modules and focusing on *BrGeBP* genes in *Brassica rapa,* emphasizes the crucial role of small RNAs in developmental transitions under stress conditions (contribution 7). As highlighted by research on RNA metabolism and transcriptional flexibility, stress adaptation involves dynamic changes across the gene expression landscape rather than the static activation of stress-inducible genes.

## 3. Hormone Signaling as a Central Integrative Hub

Phytohormones play essential roles in linking environmental signals and coordinating plant stress responses. Hormone signaling pathways, including those of ABA, cytokinin (CK), and brassinosteroid (BR), regulate many physiological processes related to stress tolerance [29,30,31].

Several studies in this Special Issue demonstrated how hormonal regulation contributes to plant abiotic-stress adaptation by mediating adjustments in metabolism and changes in gene expression. ABA-mediated signaling cascades in *Solidago gigantea* have been shown to control stomatal behavior, osmotic adjustment, and stress-responsive gene expression under extreme light, thermal, and soil conditions (contribution 8). Another study demonstrated that type B response regulator (RRB) genes are involved in responses to CK, ABA, and methyl jasmonate, as well as abiotic stresses such as drought and cold, in members of the Poaceae family, including *Oryza* (rice), *Panicum*, *Sorghum*, *Setaria*, *Zea* (corn), and *Triticum* (wheat) (contribution 9). BRs also play a protective role in plant stress adaptation, with experimental evidence indicating that treatment with these hormones enhances rice tolerance to gamma irradiation by improving its antioxidative activity and stabilizing cellular metabolism (contribution 10). Collectively, these findings highlight the importance of hormone signaling networks as central regulators of plant stress adaptation.

## 4. Cellular Homeostasis, Organelle Function, and Metabolic Reprogramming

Molecular regulatory processes ultimately converge at the cellular and physiological levels, where redox regulation plays a key role in plant response to abiotic stresses [32]. Several studies in this Special Issue highlight how ROS, antioxidative systems, and stress-priming mechanisms regulate oxidative balance and contribute to plant stress adaptation.

As highlighted by one review article in this Special Issue, ROS are natural byproducts of plant metabolism and play a central role in the maintenance of redox homeostasis. They also function as signaling molecules, in that ROS waves generated under abiotic stress trigger transcriptomic and metabolomic reprogramming, thereby promoting plant acclimation (contribution 11).

This Special Issue highlights priming-based stress acclimation as a key mechanism in stress adaptation. For example, thermopriming can temporarily boost the resilience of plants to subsequent salt stress by increasing the accumulation of protective secondary metabolites (contribution 12).

In one research paper in this Special Issue, ascorbic acid, a non-enzymatic antioxidant, was shown to enhance the activity of antioxidative enzymes, increase the levels of sugars, proline, and ABA, and protect the photosynthetic system, thereby conferring cold tolerance to tomatoes (contribution 13). Additionally, metals such as aluminum and cadmium cause cellular damage by inducing ROS accumulation (contribution 14–15). Interestingly, post-translational protein modifications, such as ADP–ribosylation, contribute to the oxidative stress response by facilitating ROS scavenging in *Nicotiana benthamiana* (contribution 16). These system-level studies link gene regulation to whole-plant performance, highlighting that an understanding of regulatory networks requires consideration of physiological outcomes.

## 5. System-Level Integration and Translational Perspectives

In contrast to previous component-based analyses, network-centered frameworks are a key feature of modern studies on stress biology [11,33]. One study in this Special Issue used transcriptomics and metabolomics to identify important regulatory hubs and interaction modules that coordinate the transcriptional, hormonal, and metabolic pathways involved in heat-stress adaptation in avocado (contribution 17).

The knowledge gained from analyzing regulatory networks can significantly enhance our understanding of post-translational regulatory processes. One review article in this Special Issue provides an overview of how protein modification mediated by kinases and phosphatases regulates various plant processes, including growth and development, hormone responses, and reactions to both abiotic and biotic stresses. Cross-species functional validation further supports the use of network-informed strategies (contribution 18). These advances indicate how a system-level understanding can drive a shift toward predictive, design-based crop improvement.

## 6. Conclusions and Future Directions

Collectively, the contributions to this Special Issue center on the theme that abiotic stress responses in plants are governed by complex, layered regulatory networks. Transcription factors, miRNAs, hormone signaling pathways, and metabolic adjustments are interconnected components of an integrated adaptive system. Understanding these interactions requires both detailed molecular analyses and comprehensive approaches to examining regulatory dynamics across multiple levels.

Despite the significant progress made in elucidating the molecular mechanisms underlying plant stress responses, key challenges remain. Understanding tissue-specific regulatory mechanisms, stress-response timing, and the interaction among multiple stresses requires further investigation. Moreover, connecting laboratory discoveries to field performance is crucial for applying these mechanistic insights to the development of agricultural crops with improved stress resilience.

By presenting a compilation of studies on molecular regulation, cellular homeostasis, and system-level integration, this Special Issue provides a comprehensive foundation to enhance our understanding of the regulatory mechanisms and gene networks that support plant adaptation to abiotic stress. We hope that the insights presented herein will motivate further research on stress response networks and facilitate the development of crops capable of thriving under increasingly challenging environmental conditions.
